# Potential benefit of β-glucans as adjuvant therapy in immuno-oncology: a review

**DOI:** 10.37349/etat.2021.00036

**Published:** 2021-04-30

**Authors:** Valeria Cognigni, Nicoletta Ranallo, Francesca Tronconi, Francesca Morgese, Rossana Berardi

**Affiliations:** Clinical Oncology, Università Politecnica delle Marche, AOU Ospedali Riuniti, 60126 Ancona, Italy; University of Southampton, UK

**Keywords:** β-glucans, mushrooms, immuno-oncology, immune-modulating, immune checkpoint inhibitors

## Abstract

Fungal compounds have long been used for centuries as food supplements. β-glucans have been identified as the most interesting molecules with beneficial effects in several chronic diseases. *In vitro* studies have shown that they are able to elicit the immune cells maturation and activation with the result of an increased release of proinflammatory cytokines and chemokines and a stimulation of anti-bacterial activity of macrophages and neutrophils. As β-glucans enhance pathogen elimination through non-self antigens identification, they can also direct immune response against tumor cells. These compounds also stimulate the activity on adaptive immune cells and they have been regarded as biological response modifiers. In this way, β-glucans can be exploited as adjuvant cancer therapy, in particular by a synergic action with chemotherapy or immunotherapy. In the immuno-oncology era, the need is to identify innovative drugs that can simultaneously target and inhibit different biological processes relevant for cancer cells survivors. Recent clinical studies showed promising results about the combination of β-glucans and immune checkpoint inhibitors for patients affected by different solid tumors. This review aims to investigate molecular mechanisms of action of β-glucans and is focused on their application in clinical practice as immune-adjuvants for treatment of cancer patients.

## Introduction

Mushrooms have been utilized as food supplements for several centuries and their therapeutic properties on human health have long been studied.

β-*D*-glucans are polysaccharides found as cell wall components with structural function and are extracted from different species of yeast, mushroom, bacteria and barley. β-glucans extracted from oat or barley have to be distinguished from ones extracted from fungal cell components, because they have different molecular structure and play distinct roles in regulation of human metabolism [1].

There are numerous pharmaceutical products based on these polysaccharides, such as schizophyllan, lentinan, grifolan, polysaccharide-peptide (PSP) complex and polysaccharide-protein (PSK) complex [2].

These compounds are known to have an immune-modulating action capable of stimulating the immune system’s response by activation of macrophages, phagocytosis of the pathogen and release of proinflammatory cytokines [3]. They positively influence the homeostasis of the organism, particularly modifying the intestinal microbiota [4–7].

Glucan receptors are expressed on macrophages, natural killer (NK) cells and neutrophils [8]. Among these dectin-1, complement receptor 3 (CR3), lactosylceramide (LacCer), natural cytotoxicity receptor p30 (NKp30) and scavenger receptors are the most studied ones [9]. β-glucans have a distinct affinity with these receptors according to the different chemical structure and are therefore capable of triggering different host responses [10].

This immune-stimulating capacity of β-glucans makes wide and varied medical use. The ability to modulate humoral and cellular immunity can be exploited, for example, in the treatment of various chronic inflammatory diseases.

β-glucans are able to reduce the level of total serum cholesterol and glucose, in addition to acting on the control of body weight [11]. The mechanism that rules the cholesterol-lowering effects of β-glucans takes place through the gut microbiota and the production of short-chain fatty acids (SCFAs, for example propionate). The gut microbiota degrades the fibers from which SCFAs are obtained; the increase of them at the expense of the acetic acid (main substrate for cholesterol biosynthesis) results in decrease in cholesterol biosynthesis [12]. Recent studies suggested that β-glucans control circulating lipid levels by excretion of fecal lipids and by regulation of the activity of hydroxy-3-methyl-glutartyl-coenzyme A reductase [13].

The β-glucans are able to attenuate blood postprandial glycemic and insulin peak forming a barrier in the small intestine which prevents glucose absorption. Moreover, the latest evidence points to a role in activating metabolic pathways through phosphatidylinositol 3-kinase (PI3K)/serine-threonine kinase (Akt), which have a key role in the pathogenesis of diabetes [14]. Furthermore the enrichment of food with β-glucan is required in order to produce low glycemic index meals suitable for diabetic subjects [15, 16].

In this way, β-glucans have a potentially beneficial activity in the prevention and treatment of diabetes mellitus, hypercholesterolemia and associated cardiovascular diseases [14, 17].

β-glucans have been studied as potential adjuvant agents in treatment of gastrointestinal, hepatic and respiratory infections, caused by bacterial, viral and fungal microorganisms.

β-glucans have been also studied as modulators of human immunodeficency virus (HIV)-associated immune dysfunction. In fact, they are involved in regulation of gut barrier permeability [18] and might be responsible for microbial translocation from the gastrointestinal tract into systemic circulation. It has been supposed an interesting role of β-glucans in the pathogenesis of non-acquired immunodeficiency syndrome (non-AIDS) events, but further studies are needed to explore their contribution in HIV infection and course [19–21].

In Eastern world, mushrooms are widely used as medical and nutritional support in cancer patients, in order to improve fatigue and cachexia and to increase tolerability to chemotherapy. In fact, numerous animal and human studies have shown remarkable activity against a wide variety of tumors [22, 23]. In the oncological field, β-glucans can stimulate the innate and adaptive immune response, inhibit the proliferation of cancer cells, promote apoptosis and block the angiogenesis [24–26].

The advantages of β-glucan derive from its non-toxicity and a non-immunogenicity due to its absence of proteins and peptide components; in this way the β-glucans have a specific modulatory activity of the immune system as they bind specific receptors [27].

The purpose of this review is to deepen the molecular mechanism of β-glucan and in particular its role as immune-modulator, the potential association with immunotherapy and future therapeutic applications.

## Benefit from β-glucans in cancer patients

As we know, the immune system is classified into adaptive and innate [28]. The first is represented by a rapid line of defense against pathogens while the adaptive system develops a long-lasting response that protects the organism from subsequent encounters with the same pathogen and therefore plays a fundamental role in the protection against infections and in the efficacy of vaccines [29–31]. One of the mechanisms that stimulate the induction of trained immunity is epigenetic reprogramming: after stimulation with certain ligands such as β-glucan or Bacille Calmette-Guerin (BCG), the immune cells undergo a functional reprogramming which involves an increase of their reactivity to the next stimulation [32]. The control and surveillance of tumors involves an intricate dance between the adaptive and innate immune system.

It has been demonstrated that the association of β-glucans with chemotherapy is able to enhance cytotoxicity and can improve patient clinical outcome. The use of medical mushroom extracts has been studied in association with chemotherapy in different kinds of cancers, such as estrogen receptor negative human breast cancer, gastric and colorectal cancer, non-small-cell lung cancer (NSCLC) and hematologic diseases [33–36].

The advent of immunotherapy has dramatically changed cancer treatment. In particular, the use of immune checkpoint inhibitors (ICIs) has had great success in the treatment of numerous types of malignancies and their use in clinical practice is progressively increasing. The response to immunotherapy is variable often due to a different involvement of the tumor microenvironment (TME). To modify TME with increasing the presentation of antigens by the tumor mass and thus stimulating the response of the immune cells towards the tumor cells is, currently, under investigation [27].

In this regard, β-glucan molecules are a potential immune-modulator that acts on the innate and adaptive immune response within TME. B-glucan could have an adjuvant role in stimulating and improve clinical response to ICIs [37].

## Molecular composition and modulation of immune cells

β-glucans are polysaccharides composed of *D*-glucose monomers linked together by 1→3 linear β-glycosidic bonds. The glycosidic chain core has 1→6 side branches that are specific of fungi-derived glucans [38]. In some subgroups of fungi, the polysaccharide chain may also be bound with protein or peptides, by forming PSK or PCP complexes [39].

β-glucans has been deeply studied as biological response modifiers (BRMs) [40]: they interact directly with receptors located on plasmatic membrane of immune cells and are able to elicit an effective inflammatory and immune response against non-self antigens expressed by pathogens but also on tumor cells [41].

The immune-modulatory properties of β-*D*-glucans have been widely investigated and it has been supposed that they exert an antitumor activity, by enhancing immune system against tumor cells and by inhibiting tumor invasion and progression through a complex modulation of mechanisms of apoptosis and angiogenesis [42].

**Figure 1.**
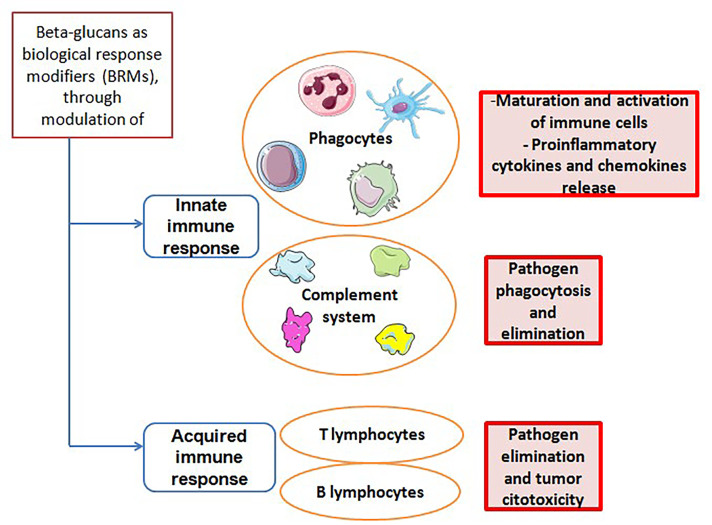
Interaction pathways between β-glucans and the immune system

Several preclinical and clinical studies have shown that β-*D*-glucans are able to modulate the responsiveness and the interaction between innate and adaptive immune systems [43] (Figure 1). They can enhance antimicrobial activity of macrophages, monocytes and neutrophils, leading to maturation of these target cells and to an increased proinflammatory cytokine and chemokine release. This reflects in a stimulation of adaptive immune cells, including CD4^+^ T cells, CD8^+^ cytotoxic T lymphocytes (CTL) and B cells. Specifically, the tumoricidal mechanisms carried out by T cells and induced by β-glucans can be summarized in this way. The β-glucan binds specific receptors (for example, Dectin-1 which will be discussed below) expressed on myeloid cells, which are converted into antigen presenting cells (APCs). The binding activates the CD4^+^ and CD8^+^ T cells which in turn will produce pro-inflammatory cytokines [respectively tumor necrosis factor (TNF)-α, anti-tumor cytokine interferon-gamma (IFN-γ), Granzyme B, and perforins], leading to the destruction of cancer cells [44]. Similarly β-glucan induces switching of suppressive M2 macrophages into inflammatory M1 macrophages which in turn will activate Th1-type T cells, causing damage to cancer cells through the secretion of pro-inflammatory cytokines by T cells [45]. On the other hand, the link between β-glucan and polymorphonuclear cell will cause cell apoptosis with the release of ROS in the microenvironment leading to the death of tumor cells from oxidative stress [46]. Besides, β-*D*-glucans are also modulators of NK cells cytotoxicity. These compounds are able to stimulate activation of NK cells against tumor cells, both by production and release of pro-inflammatory cytokines and by complement activation [47]. In this context, it has been shown that β-glucans can induce granulopoietic progenitors and, in general, innate immune cells towards a tumor-suppressive phenotype [48].

In this way, it has been hypothesized that β-*D*-glucans may play an important role as immune-modulator agents and can be used as a synergic treatment in combination with ICIs.

## Receptors of β-glucan

The interaction between β-glucans and their receptors on human cells is likely to be very complex and only partially known. The main receptors that are involved in inflammatory and immune-response are Dectin-1, CR3, LacCer, NKp30 and scavenger receptors [9].

### Dectin-1

Dectin-1 is a type II transmembrane pattern-recognition receptor with an extracellular C-type lectin-like domain fold and a cytoplasmic domain that contains an immunoreceptor tyrosine-based activation motif (ITAM) [10, 49]. After binding of β-glucans, it can mediate the non-opsonic phagocytosis of opportunistic pathogens and it is responsible of cytokine release [50]. In fact, Dectin-1 induces phosphorylation of its ITAM and spleen tyrosine kinase (Syk) and activates an intracellular PI3K/Akt pathway. Thus, the result is an increased production and release of various inflammatory cytokines, such as TNF, CXC-chemokine ligand 2 (CXCCL2), Interleukin-2 (IL-2), IL-10 and IL-12. Dectin-1 can also collaborates with Toll-like receptors (TLRs) expressed on the same cell, in order to enhance cytokine production, such as TNF-α, IL-6, IL-10 and IL-23, and down-regulate release of IL-12 [51].

Furthermore, Dectin-1 associated intracellular signaling also involves cytoplasmic Nod-like receptor protein 3 (NLRP3) inflammasome, which activation seems to be essential for IL-1b production and secretion [52]. In human cells, Dectin-1 has another binding site that can recognize an endogenous ligand on T cells: in this sense, Dectin-1 has been proposed to act as a T cell co-stimulatory molecule [53].

### CR3

CR3, also known as macrophage 1 antigen (Mac-1), belongs to the family of β2 integrins and is an heterodimeric glycoprotein composed of two non-covalently associated chains (CD18 and CD11b), found on immune cells such as neutrophils, macrophages, lymphocytes and NK cells [54]. Its lectin-like domain is responsible for binding to β-glucans and this binding primes leukocytes for cytotoxicity and phagocytosis of target cells, through a Syk-PI3K molecular pathway. In fact, CR3 has a second ligand able to recognize iC3b-coated tumor cells and its activation for complement-dependent cytotoxicity and then tumor cell lysis requires its dual binding to iC3b and β-glucan [55, 56].

### LacCer

LacCer is the most abundant neutral glycosphingolipid and it is expressed on various human cells, among these on neutrophils. LacCer forms lipid rafts on plasmatic membrane of neutrophils and, after binding with β-glucans, it activates a signal transduction pathway involving Src family kinase/PI3K. The result is neutrophil chemotaxis [57, 58] and enhanced cytokine release [59, 60]. Furthermore, it has been demonstrated that CR3 and LacCer was partially co-localized on lipid rafts of plasma membrane of neutrophils and some preclinical data suggest that LacCer-mediated phagocytosis may be dependent on CR3, suggesting a co-stimulatory activity of these two β-glucans receptors [61].

### NKp30

NKp30 belongs to immunoglobulin-like transmembrane receptor family and it was needed to bind 1-3 β-glucan by NK cells. It has been demonstrated that this receptor can activate Src family kinases and mediate granule polarization and perforin release in NK cells. In this sense, NKp30 has been recognized as a pattern-recognition receptor, whose stimulation enhances NK cell killing of fungi, such as *Cryptococcus neoformans* and *Candida albicans* [62].

### Scavenger receptors

It has been demonstrated that fungal β-glucans can also bind to other membrane receptors, such as scavenger receptors. β-glucans can interact with the CD5 ectodomain, present on plasmatic membranes of T and B cells and can induce mitogen-activated protein (MAP) kinase activation and cytokine release [63].

### Preclinical studies

Several β-glucans extracted from different species of fungi have been investigated as BRMs and the most relevant ones are described in Table 1.

**Table 1.** Biological activity of most relevant β-glucans investigated in pre-clinical studies

**Pre-clinical study (ref.)**	**Treatment models**	**β-glucans/fungus**	**Biological activity**
Sorimachi K, et al., [64] Niu YC, et al., [65]	Animal model (rat bone marrow) Animal model (mouse S180 cells), IV administration	Glucan/*Agaricus blazei Murill*	- ↑ secretion of IL-8 and TNF-α by macrophages - ↑ production of IL-23, IL-12, IL-1 - ↑ cytokine and leukocyte growth factor production
Kubala L, et al., [66] Zhong K, et al., [67]	Human model Mice splenic Lymphocytes	Schizophyllan/*Schizophyllum commune*	- ↑ lymphocytes proliferation, through production of IL-2 - ↑ production of pro-inflammatory cytokines IL-6, IL-8, and TNF-alpha
Yang A, et al., [68]	Murine macrophages Human hepatoma HepG2 cells	TPG-1/*Trametes robiniophila*	- ↑ production of TNF-α and IL-6 through toll-like receptor 4 (TLR4)
Wang J, et al., [69] Wang SY, et al., [70] Chien CM, et al., [71]	Animal model, *in vitro*, Raw 264.7 cells Human myeloid leukemia cell lines Human umbilical cord blood	Proteoglycan fraction/*Ganoderma lucidum*	- ↑ anti-inflammation activity against lipopolysaccharide (LPS) stimulation - ↑ T cells activity - ↑ expression of IL-1, IL-2, IL-6, TNF-α and INF-γ - modulate NK cell cytotoxicity
Carmona EM, et al., [72] Ali MF, et al., [73]	Rat cells Human model	Glucan/*Pneumocystis* and *Aspergillus*	- ↑ TNF-α, IL-6 and IL-8 production by B-lymphocytes - ↑ IL-23 and IL-6 release by dendritic cells - ↑ secretion of IL-17 and IL-22, both Th17-produced cytokines
Kankkunen P, et al., [52] Ding J, et al., [74] Vetvicka V, et al., [47]	Human macrophages Human monocyte-derived DCs Murine melanoma B16 cell line	Glucan/*Saccharomyces cerevisiae*	- ↑ Th2 immune response and inhibits Th1 by promoting the release of anti-inflammatory cytokines such as IL-10 and transforming growth factor (TGF-β) - ↑ IL-1β transcription and secretion - ↑ IL12, IL-2, TNF, IFN production - ↑ tumor-specific CTL activity - modulate NK cells activity and killing towards tumor cells
Ina K, et al., [75]	Human model (gastric cancer patients), IV administration	Lentinan*/Lentinula edodes*	- ↑ PD-L1 tumor expression - ↑ release of TNF-α, IL-12 and IFN-γ - ↑ T cells activity, especially CD8^+^ cells and Th1 polarization
Masuda Y, et al., [76] Masuda Y, et al., [77]	Animal model Animal model	Maitake α-glucan (YM-2A)/*Grifola frondosa*	- ↑ activation of dendritic cells and macrophages - ↑ IFN-γ production by CD4^+^ and CD8^+^ T cells
Bose N, et al., [78]	Human serum	Imprime PGG/*Saccharomyces cerevisiae*	- ↑ phenotypic and functional activation of monocytes - ↑ activation of neutrophils, through production of IL-8, CCL2, and CD11b

We focused on BTH-1677 (Imprime-PGG) because it is one of the most studied β-glucans in combination with ICIs.

BTH-1677 is a water-soluble and intravenous formulation of yeast-derived β-glucan purified from the cell wall of *Saccharomyces cerevisiae*. It is recognized as a fungal pathogen-associated molecular pattern (PAMP) and it is able to trigger a complex and coordinated immune response that involves both innate and adaptive immune cells. Its mechanisms of action as immune-modulator and anti-tumoral agent has been deeply investigated in preclinical and clinical studies.

Bose et al. [78] demonstrated that BTH-1677 interacts with human-isolated neutrophils and monocytes through a CR3 and complement-dependent manner. This result has been also confirmed in a mice model [79].

As shown in preclinical studies [80], when BTH-1677 enters the blood, it is bound by endogenous plasma anti-β-glucan antibodies (ABAs) and it constitutes a tripartite immune complex, by attracting the opsonization by complement protein iC3b. This macro-complex interacts with CR3 and FCγ receptor II (CD32a) located on neutrophils, macrophages and monocytes, and stimulates inflammatory cytokine production and release. It results in activation of innate immune response and enables direct killing of antibody-targeted tumor cells, through a mechanism of antibody-dependent cellular phagocytosis [81, 82]. Dectin-1 binds directly BTH-1677 and other fungal compounds, but it has been suggested that the tripartite immune complex described above could also signal through Dectin-1, by interacting simultaneously with CR3 [81]. These innate immune functions are detected only in the presence of sufficient ABA in serum. It suggests that ABA levels may be a biomarker to select patients who have benefit to administration of β-glucans and that the exogenous supplementation of ABA could apport a rehabilitation of these functions [83, 84].

BTH-1677 can affect macrophage differentiation toward M1 phenotype respect than the suppressive M2 state [85, 86] and inhibits activation of myeloid derived suppressor cells (MDSC) against T cell proliferation [87]. Chan et al. [86] demonstrated that BTH-1677 can modulate *in vitro* activity of monocyte-derived dendritic cells (MoDCs), by eliciting an increased surface expression of the maturation and co-stimulatory markers, such as CD80, CD83, CD86 as well as HLA-DR. It leads to an increased production of anti-tumor cytokine IFN-γ and surface expression of antigen presentation markers. Furthermore, among biological effects induced by BTH-1677, it seems to be that it up-regulates programmed death-ligand 1 (PD-L1) expression on macrophages surface and also on tumor cells [88].

The activity on innate immunity is also expressed on NK cells: BTH-1677 enhances NK cell functionality and killing, which are dependent on IFN-γ and Dectin-1 and represent the main antibody-dependent cellular cytotoxicity against tumor cells [89].

Besides, BTH-1677 can inhibit the suppressive activity of Tregs on CD4^+^ T cells and is able, when treated with whole blood, to enhance CD4^+^ and CD8^+^ T cell proliferation, also by driving T cell polarization towards anti-tumor Th1 phenotype [90, 91].

In order to exploit the immunomodulating effect of BTH-1677, several preclinical studies investigated the combination of this β-glucan with anti-tumor monoclonal antibodies (mAbs) acting on immune response. The aim was to improve the efficacy of ICIs, in particular anti-programmed death receptor-1 (anti-PD-1)/ PD-L1 mAbs, in combination with β-glucans, by inducing PD-L1 tissutal expression and by enhancing a potent and coordinated immune response against tumor cells.

Fraser et al. [92] demonstrated in two distinct mice models (CT-26 and MC-38 model) that the combination of BTH-1677 with anti-PD-1 or anti-PD-L1 agents is able to repress tumor growth, compared to models treated only with BTH-1677 or anti-PD-1/anti-PD-L1.

In another study, Qiu et al. [93] showed that T cells treated *ex vivo* with anti-PD-1 antibody and co-cultured with BTH-1677 treated macrophages or dendritic cells have an increased proliferation rate. Then, they observed a significantly reduced median tumor volume in a mice model after administration of both BTH-1677 and anti-PD-1 mAbs.

In summary, what emerged is how the monocyte-macrophages cells activated by BTH-1677 *in vitro* have an higher expression of membrane PD-L1; simultaneously, an increase in the expression of the co-stimulatory molecule CD86 and other cytokines has been documented, which allow further stimulation on the effector action of T cells. Furthermore, the tumor cells themselves, thanks to the activation of a wider, innate and adaptive immune response induced by BTH-1677 show an increased expression of PD-L1.

Another interesting data reported is that BTH-1677 has the ability to turn off the inhibitory effect of PD-L1 upregulation by enhancing the secretion of immunostimulatory cytokines and the expression of costimulatory proteins. These data confirm the idea that BTH-1677 synergizes with ICIs, particularly with anti-PD1 antibodies, and it may represent an effective adjuvant therapy along with approved and widely used immunotherapy agents [94].

**Figure 2.**
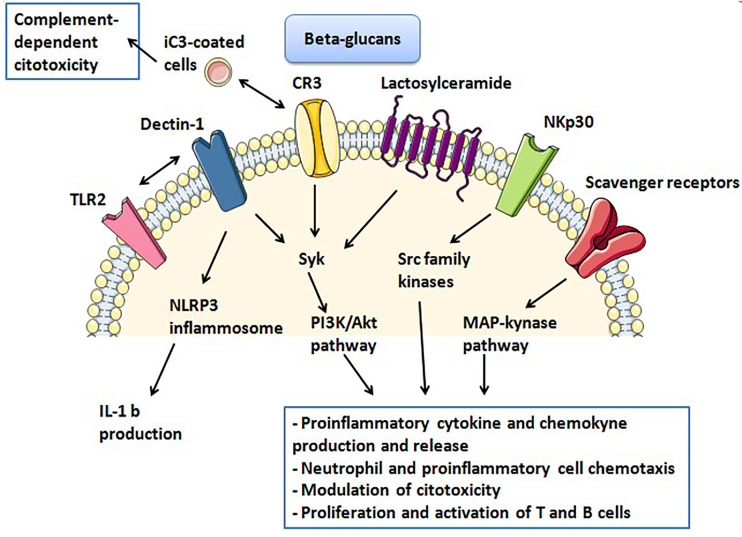
Activation of β-glucan receptors and intracellular pathways. TLR2: toll-like receptor 2

In a similar way, Hong et al. [95] have demonstrated the same effect of a combined therapy of β-glucan and anti-tumor mAbs. In this study developed on mice models, the combining administration of β-glucan and mAbs against naturally occurring antigens GD2 ganglioside or recombinant human Mucin1 (MUC1) elicited a significantly greater tumor regression, respect to the mAbs or β-glucan therapy alone. In this study, the expression of CR3 on leukocytes and the binding of iC3b on tumors seem to be crucial for the β-glucan adjuvant effect and granulocytes showed to be responsible for antitumor activity (Figure 2).

## Clinical studies

Considering the promising results described above in the preclinical models, several trials evaluated the interaction of glucans with mAbs in humans (Table 2).

**Table 2.** Biological activity of most relevant β-glucans investigated in clinical studies

**Clinical trial (ref.)**	**Features**	**Patients**	**β-glucans/other drugs**	**Results**
Halstenson CE, et al., [96]	Phase 1a/b. Single center, randomized, double-bind, placebo-controlled, dose escalation study	Healthy volunteer subjects	Imprime PGG	Acceptable safety profile, well tolerated
NCT03246685 [97]	Phase 2. Multicenter, open-label study	Advanced Squamous Cell Carcinoma of H&N (SCCHN) patients	Imprime PGG & Pembrolizumab	Terminated for enrollment failure
Uhlik M, et al., [98]	Phase 2. Multicenter, open-label study	44 ABA positive mTNBC patients	Imprime PGG & Pembrolizumab	mTNBC: - Objective response rate (ORR) was 15.9%, with 1 complete response (CR) and 6 partial responses (PR) - 17/44 shown stable diseases (SD) for more than 1 year (best response) and 4/44 for more than 2 years - Overall survival (OS) rate at 1 year was ~ 63%; median OS is 18.1 months by Kaplaian-Meyer estimation (95% CI, 12 months-not reached)
Chan A, et al., [99]	Phase 2. Multicenter, open-label study	40 ABA positive metastatic melanoma patients	Imprime PGG & Pembrolizumab	Melanoma patients: - disease control rate (DCR) was 45% (with 1 CR and 8 SD) - median OS was 8.8 months (with 12 month OS rate equal to 45%)
Modak S, et al., [100]	Phase 1.	24 chemo-resistant NB patients	Oral β-glucan & 3F8	- Acceptable safety profile, well tolerated - The maximum dose tolerated of β-glucan was not reached - A clinical response was observed in 63% of patients
Kushner BH, et al., [101]	Phase 1.	15 high-risk NB patients in remission	β-glucan & bivalent gangliosides vaccine	Acceptable safety profile, well tolerated
NCT03003468 [102]	Phase 1b/2.	mNSCLC patients	Imprime PGG & Pembrolizumab	On going
NCT03555149 [103]	Phase 1b/2. Open-label, multicenter, randomized Umbrella study	mCRC patients (cohort 3)	Imprime PGG & Atezolizumab (cohort 3)	On going

mCRC: metastatic CRC; mNSCLC: metastatic NSCLC; mTNBC: metastatic triple-negative breast cancer; NB: neuroblastoma

As aforementioned, BTH-1677 is one of most investigated compounds in the field of new frontiers in cancer therapies.

In 2015, Halstenson et al. [96] designed a single center, randomized, double-bind, placebo-controlled, dose escalation study investigating the safety and the tolerability of intravenous injection of BTH-1677 in healthy subjects. In the phase 1a, BTH-1677 was administered to 18 volunteers (≤ 45 years) sequentially randomized to receive the single dose of study drug at 0.5-1-2-4 or 6 mg/kg dose. The control group consisted of 6 people, who received a single dose of placebo. In the phase 1b, 12 subjects were randomized (3:1) to 7 daily i.v. infusion at 1, 2 or 4 mg/kg or placebo respectively. Adverse events (AEs) occurred (headache, dyspnea, paresthesia, nausea, rash and flushing) were mild and moderate, and were described in 67% of the study subjects overall. The appearance of an infusion reaction during the administration of the study product at a dosage of 4 mg/kg resulted in an amendment contemplating a slow administration of the 4 mg/kg and 6 mg/kg doses. In conclusion, the drug was well tolerated after single doses up to 6 mg/kg and after 7 daily doses up to 4 mg/kg.

The benefit of this β-glucan has been evaluated in association with pembrolizumab in various phase II studies in cancer patients. The rationale of the combination with ICIs is based on the hypothesis that glucans may stimulate immune system activation pathways complementary to that triggered by monotherapy with mAbs.

In 2017, a phase II trial aimed to investigate the benefit of this combo in head and neck cancers that failed or experienced stable disease during pembrolizumab monotherapy. Unfortunately, the study has been terminated for enrollment failure [97].

Encouraging data derived from IMPRIME 1, a phase II open-label multicenter trial involving ABA positive mTNBC and metastatic melanoma patients. In the breast cohort, 44 mTNBC pre-treated patients were enrolled to receive Imprime (4 mg/kg i.v. days 1, 8, 15 of each 3-week cycle) plus pembrolizumab 200 mg on day 1 of each cycle [98]. The results presented at San Antonio Breast cancer Symposium in 2019, showed that the primary endpoint, ORR, was 15.9%, with 1 CR and 6 PR. seventeen of 44 patients were stable for more than 1 year (best response) and 4 of 44 for more than 2 years. OS rate detected at 1 year was ~63% and median OS is currently 18.1 months by Kaplaian-Meyer estimation (95% CI, 12 months-not reached) [99]. Moreover, it demonstrates a large activation of both myeloid and T cells with extensive infiltration in tumor tissue samples [98]. These data allow to reacquire a possible role of ICIs in combination with immune-stimulating agents in patients with mTNBC, overcoming the poorer encouraging results of single agent immunotherapy in this setting [104].

In the melanoma subgroup, patients who have failed ICIs therapy undergo the same combined therapeutic regimen. The disease control rate, on 40 patients enrolled, was 45% (with 1 CR and 8 SD) and the median OS was 8.8 months (with 12-month OS rate equal to 45%). As in the breast cancer population, the indirect signs of the improved immune stimulation were observed in tissue samples (biopsy) and in peripheral blood. Therefore, in melanoma patients with disease control this biological finding is linearly correlated with a proportional increase in survival [99].

As previously explained, significant levels of circulating ABA are a sine qua non condition to expect an immune-stimulating β-glucan effect [78, 83].

In this regard, in a case-report it has been supposed that the function of β-glucan can be rehabilitated providing an intravenous supplement of purified ABA or commercial intravenous immunoglobulin G (IVIG) in deficient subjects [105].

This is a case of 84-year-old woman with pre-treated neuroendocrine tumor with baseline ABA levels < 1 μg/mL, who received BTH-1677 (4 mg/kg) in compassionate use, pembrolizumab (200 mg flat dose) and immunoglobulin G (IgG, 500 mg/kg). Immunoglobulins were infused the day before the first cycle of therapy and then on the same day of every subsequent recycle. The treatment was well tolerated, there were no AEs except a worsening of a myalgia in the first cycle. Dose and timing of administration of IVIG (1,000 mg/kg, administered on the same day of treatment, before the infusion of BTH1677 and pembrolizumab) was adapted based on the serum IgG levels detected in the pre-treatment blood samples.

This case-report showed that the minimum amount of IVIG supplement appears to be 1,000 mg/kg, based on the increase in complement activity and cytokine levels. A partial response in radiological evaluation was detected at the second cycle (reduction of 5% of target lesion); at the subsequent control (4th cycle) there was a volume increase of target lesions, no other lesions were detected.

Several phase 1 studies, which aim to investigate the role of β-glucans, have been conducted on young patients with NB, a childhood cancer still orphaned by effective therapeutic strategies. In 2013 twenty-four patients with chemo-resistant NB were enrolled to receive 10 mg/(kg·day) of 3F8 (a mAb directed against the ganglioside GD2, a NB surface antigen) associated in each cycle to an oral β-glucan, dose escalated from 10 to 80 mg/(kg·day). Most patients have a good tolerance of oral β-glucan: there were two cases of severe thrombocytopenia, of which one has not regressed and evolved into chronic. This side effect has never been described in 3F8 and could be linked to glucan mediated CR3 activation. Other AEs reported were fever, pain and urticaria (probably related to 3F8). The maximum dose tolerated of β-glucan was not reached. A clinical response, although not striking and of short duration, was observed in 63% of patients [100].

Kushner et al. [101] conducted a phase 1 study, involving 15 high-risk NB patients in second or later remission who received a combo of an immune-stimulating β-glucan and a vaccine that induced host anti-ganglioside G2/G3 antibodies. The hypothesis is that the stimulation of humoral immunity can maintain the disease remission even among these very high-risk patients. The vaccine/β-glucan treatment was well tolerated with no dose-limiting toxicity; painful local reactions have been reported immediately after the injection but no other early or delayed toxicity has been documented. The serological and minimal residual disease (MRD) responses were encouraging.

Finally, there are two interesting ongoing trials, regarding the association of ICIs and glucan.

(1) The first one (phase Ib-II trial) includes patients with mNSCLC, receiving the combination of pembrolizumab and Imprime PGG in second line. The phase II trial will test whether addition of the glucan to ICIs increases median progression-free survival (PFS) [102].

(2) In Morpheus (phase Ib-II) study, the cohort 3 composed by mCRC patients will be randomized to one of the immunotherapy combination arms, including atezolizumab and Imprime PGG, or the standard-of-care control arm. All patients included in the study are BRAF wild type or without microsatellite instability, in disease progression during or following 2 (but not more) lines of treatment for mCRC, not prior treated with ICIs [103].

## Conclusion and future perspective

The new era of drug development has seen integrative therapies as one of the options for numerous diseases. The future of cancer immunotherapy could rely on combination therapies based on ICIs with other adjuvant drugs, such as personalized cancer vaccines and targeted therapies directed to the TME. Bioactive compounds extracted from mushrooms have been shown immuno-modulator activity by stimulating the maturation, differentiation and proliferation of innate and adaptive immune cells. In addition, they are well tolerated and possess a very low risk of significant negative side effects. In this way, β-glucan demonstrate synergic effect with antitumor mAbs agents and they can have an important role to improve the therapeutic effects and the quality of life in cancer patients.

Further studies and clinical investigations are directed to evaluate how mushrooms can be used as complementary/integrative therapy to guide oncology strategies. More research and investigations are necessary to evaluate the route of administration and dosage of β-glucans and their immune effect on bacterial growth and human microbiota.

The future perspective should be also oriented to discovering molecular mechanisms of different fungal compounds, interactions with cancer immunotherapy and future applications in clinical practice.

## Abbreviations

ABAs: anti-β-glucan antibodies
AEs: adverse events
CR: complete response
CR3: complement receptor 3
ICIs: immune checkpoint inhibitors
IFN-γ: interferon-gamma
IgG: immunoglobulin G
IVIG: intravenous immunoglobulin G
LacCer: Lactosylceramide
mAbs: monoclonal antibodies
mTNBC: metastatic triple-negative breast cancer
NB: neuroblastoma
NK: natural killer
NKp30: natural cytotoxicity receptor p30
NSCLC: non-small-cell lung cancer
OS: overall survival
PD-L1: programmed death-ligand 1
PI3K: phosphatidylinositol 3-kinase
PR: partial responses
SD: stable diseases
TME: tumor microenvironment
TNF: tumor necrosis factor

## Declarations

### Copyright

© The Author(s) 2021.
